# Novel Insights into the Pathogenesis of Spinal Sarcopenia and Related Therapeutic Approaches: A Narrative Review

**DOI:** 10.3390/ijms21083010

**Published:** 2020-04-24

**Authors:** Yu-Kai Kuo, Yu-Ching Lin, Ching-Yu Lee, Chih-Yu Chen, Jowy Tani, Tsung-Jen Huang, Hsi Chang, Meng-Huang Wu

**Affiliations:** 1 School of Medicine, College of Medicine, Taipei Medical University, Taipei 11031, Taiwan; ken0975515036@gmail.com; 2 Department of Medicine, Keelung Chang Gung Memorial Hospital, Keelung 20401, Taiwan; 3 Department of Medical Imaging and Intervention, Chang Gung Memorial Hospital, Keelung & Chang Gung University, College of Medicine, Keelung 20401, Taiwan; yuching1221@gmail.com; 4 Department of Orthopedics, Taipei Medical University Hospital, Taipei 11031, Taiwan; ejaca22@gmail.com (C.-Y.L.);; 5 Department of Orthopedics, School of Medicine, College of Medicine, Taipei Medical University, Taipei 11031, Taiwan; aleckc2424@gmail.com; 6 Graduate Institute of Clinical Medical Sciences, College of Medicine, Chang Gung University, Taoyuan 33302, Taiwan; 7 Department of Orthopedics, Shuang Ho Hospital, Taipei Medical University, New Taipei City 23561, Taiwan; 8 Stanford Byers Center for Biodesign, Stanford University, Stanford, CA 94305, USA; jowytani@gmail.com; 9 Department of Neurology, Wan Fang Hospital, Taipei Medical University, Taipei 11696, Taiwan; 10 Ph.D. Program for Neural Regenerative Medicine, College of Medical Science and Technology, Taipei Medical University and National Health Research Institutes, Taipei 11031, Taiwan; 11 Department of Pediatrics, School of Medicine, College of Medicine, Taipei Medical University, Taipei 11031, Taiwan; 12 Department of Pediatrics, Taipei Medical University Hospital, Taipei 11031, Taiwan

**Keywords:** image detection, muscle, myogenesis, pathogenesis, spinal sarcopenia

## Abstract

Spinal sarcopenia is a complex and multifactorial disorder associated with a loss of strength, increased frailty, and increased risks of fractures and falls. In addition, spinal sarcopenia has been associated with lumbar spine disorders and osteoporosis, which renders making decisions on treatment modalities difficult. Patients with spinal sarcopenia typically exhibit lower cumulative survival, a higher risk of in-hospital complications, prolonged hospital stays, higher postoperative costs, and higher rates of blood transfusion after thoracolumbar spine surgery. Several studies have focused on the relationships between spinal sarcopenia, appendicular muscle mass, and bone-related problems—such as osteoporotic fractures and low bone mineral density—and malnutrition and vitamin D deficiency. Although several techniques are available for measuring sarcopenia, each of them has its advantages and shortcomings. For treating spinal sarcopenia, nutrition, physical therapy, and medication have been proven to be effective; regenerative therapeutic options seem to be promising owing to their repair and regeneration potential. Therefore, in this narrative review, we summarize the characteristics, detection methodologies, and treatment options for spinal sarcopenia, as well as its role in spinal disorders.

## 1. Introduction

Spinal sarcopenia—the loss of skeletal muscle mass and function—is of crucial concern, owing its the high rate of adverse outcomes among older adults [1]. Skeletal muscle strength declines by the age of 40 years in both sexes [1]. Muscle power declines approximately 10% more than muscle strength in men, whereas no significant differences are observed in women [1]. In some articles, it has been reported that handgrip strength declines faster in older women compared with older men [2,3]. A study from Korea revealed that in addition to advanced age, crucial factors associated with sarcopenia in all age groups were physical activity, blood pressure, waist circumference, and triglyceride and vitamin D levels [4]. Notably, overall energy intake was related to sarcopenia among young adults, whereas fasting glucose, suicidal ideation, and sex were factors related to sarcopenia among the elderly. In addition, Brzeszczyńska J. et al. found that increased cellular stress, with impaired oxidative stress and misfolded protein response, were associated with the development of sarcopenia based on the in vitro system of myogenesis [5].

Notably, the prevalence of spinal sarcopenia varies in different regions worldwide [6,7,8,9,10]. For one systemic review, a meta-analysis was conducted, and the overall prevalence was estimated to be 10% (95%, confidence interval (CI): 8–12%) in men and 10% in women (95%, CI: 8–13%) [11]. Moreover, the results demonstrated that the prevalence was higher among non-Asian individuals than in Asian individuals of both sexes [11]. Another study indicated that estimates of sarcopenia prevalence vary widely because of the definitions used (e.g., those of the European Working Group on Sarcopenia, the Asian Working Group on Sarcopenia, the International Working Group on Sarcopenia) and appendicular lean mass or weight definitions [12]. Sarcopenia is often associated with various pathologic conditions, such as Alzheimer’s disease and rheumatoid arthritis in women, both of which increase the need for institutional care as well as the mortality rate of hospitalized older adults [13,14,15,16,17]. A cohort study for estimating the mortality risk revealed that muscle strength is a better marker of muscle quality than muscle quantity, and grip strength provides risk estimates similar to those of quadricep strength [18]. Furthermore, the researchers of several studies have recognized sarcopenia as an independent predictor of overall survival among patients undergoing surgeries such as radical cystectomy for bladder cancer and the resection of pancreatic adenocarcinoma [19,20].

While the pathophysiology of spinal sarcopenia is complex, several studies have discussed the mechanism which leads to skeletal muscle atrophy, including insulin resistance, myostatin activation, mitochondrial function, and glucocorticoid response [21,22,23,24,25]. Regarding the relationship between sarcopenia and obesity, elevated insulin resistance—which results in obesity and metabolic syndrome—was found in patients with sarcopenia, probably because of their reduced available insulin-response muscle [21]. Besides this, increased fat mass accompanied with obesity provoked the production of tumor necrosis factor-α, interleukin-6, and other adipokines which further promote insulin resistance and have a catabolic effect on muscle [26]. In the end, a vicious cycle is hence created. Myostatin, which is growth differentiation factor 8, is considered to be another contributor to sarcopenia-related obesity in the transforming growth factor-β superfamily. It is found in abundance in skeletal muscle and less in adipose tissue as well as cardiac muscle [27,28]. In the study performed by Yarasheski KE et al., their results found that serum myostatin increased with aging, which suggested that human serum myostatin might be a biomarker of age-related sarcopenia [29]. The Baltimore Longitudinal Study suggested that skeletal muscle ex vivo mitochondrial respiration parallels decline in vivo oxidative capacity, cardiorespiratory fitness, and muscle strength [30]. This finding was consistent with many human studies, which have shown that mitochondrial function declines with age, perhaps due to the exaggerated apoptotic sensitivity found in the elderly that hence leads to age-related sarcopenia [31,32,33,34]. Glucocorticoids also provide an important influence on muscle atrophy. Glucocorticoid-induced muscle atrophy is based on the activation of the ubiquitin proteasome and the lysosomal systems, which leads to muscle proteolysis. In addition, these two systems were confirmed to be mediated by the increased expression of several atrophy-related genes, such as FOXO and Atrogin-1 [35].

Studies have demonstrated the role of sex in spinal sarcopenia [36,37]. Sylvia Kirchengast et al. conducted a population-based study to analyze gender differences in the prevalence of sarcopenia among elderly [37]. The results showed that sarcopenia was found more frequently among women in the youngest age group (<70 years), while the opposite was true in the oldest age group (>80 years) [37]. Besides this, L. Tay et al. also demonstrated sex-specific pathophysiological mechanisms for sarcopenia, which may be applied to clinical intervention in patients with sarcopenia in both sexes [36]. The results showed that malnutrition and higher triglyceride levels appear to be risk factors for sarcopenia in women and men, respectively [36]. On the other hand, therapies that block myostatin signaling may be relevant in male elderly patients, while the role of IGF-1 agonists may hold greater promise in female elderly patients [36]. Despite numerous studies having confirmed the benefit of testosterone in treating sarcopenia, including increased muscle mass and grip strength, the potential risks cannot be neglected [38,39,40]. The risks of testosterone supplementing include sleep apnea, thrombotic complications, and prostate cancer [41]. Associated with a decline in estrogen levels, the menopause also leads to decreased muscle mass and muscle strength [42].

In recent years, several original articles have discussed how spinal sarcopenia influences spinal parameters in individuals, including cervical lordosis, thoracic kyphosis, and lumbar lordosis [43,44,45,46]. Notably, these spinal changes often lead to pathologic conditions such as degenerative scoliosis and compression fractures which are associated with poor life quality compared to their normal counterpart [46]. However, only a few studies have examined spinal sarcopenia and its pathogenesis comprehensively. Hence, this review incorporates these findings to provide new evidence regarding the mechanisms, evaluating methods, and treatment modalities for spinal sarcopenia.

## 2. Progression and Characteristics of Spinal Sarcopenia

Spinal muscle volume significantly influences spinal disorders in terms of pain, life quality, and spinal function [46]. Studies have determined that the loss of muscle mass owing to aging can lead to an imbalance between the extensor and flexor muscles of the spine [47,48,49]. This imbalance associated with spinal sarcopenia, along with all its other alterations, is noted in various parts of the spine. Moreover, this phenomenon could be caused by degenerative scoliosis followed by a forward bending of the trunk, owing to the specific myopathy of the paraspinal muscles [48]. Furthermore, the cross-sectional area (CSA) of the paraspinal muscles tends to decrease with age; the fat infiltration rate also increases with age [50]. Hence, it is crucial for patients with lumbar scoliosis to have their spinal sarcopenia treated and alleviate muscle loss [51]. It has been reported that in patients with chronic low back pain, their CSA is smaller and their fat tissue increases [52]. Moreover, multifidus atrophy typically leads to the loss of lumbar segmental stability, resulting in a high rate of low back pain recurrence [53]. Regarding middle-aged patients with chronic low back pain, studies have revealed that CSA and relative CSA (rCSA: CSA ratio of muscle and disc) influence the overall strength of back muscles and back flexors [54]. In general, multiple factors contribute to degenerative spinal sarcopenia, including spinal muscle imbalance and back muscle atrophy.

Toyoda et al. conducted an observational study to demonstrate the correlations between back muscle strength, trunk muscle mass, handgrip strength, and gait speed and sarcopenia-related parameters in patients with spinal disorders [43]. Of these, the trunk muscle mass, handgrip strength and gait speed were all found to be significantly correlated with back muscle strength in both genders. Spinal disorders might be one of multiple risk factors for spinal sarcopenia, which is considered to be a geriatric disease. Another study analyzed how spinal sarcopenia affects degenerative lumbar scoliosis (DLS) and lumbar spinal canal stenosis (LSCS) [44], and revealed more sarcopenia complications in patients with DLS compared with those with LSCS (46.6% compared with 16%). Moreover, both appendicular and truncal skeletal muscle mass indexes (SMIs) were lower in patients with DLS. That study demonstrated that spinal sarcopenia might be involved in the pathogenesis of scoliosis. Appendicular skeletal muscles were related to posterior pelvic tilt, whereas the truncal muscles affected stooped posture, posterior pelvic tilt, lumbar scoliosis, and vertebral rotation. The study’s results suggested that a decrease in pelvic or lumbar support structures, such as truncal and appendicular muscle mass, may be involved in the progression of spinal deformities and increased lower back pain. Notably, a decrease in truncal muscles was confirmed as a significant risk factor for DLS regardless of age [46]. A case-control study determined that, compared with the control group, the prevalence of spinal sarcopenia was significantly higher in the DLS group (24% compared with 12%) [55]. Furthermore, in the DLS group, the spinal sarcopenia subgroup exhibited inferior results in a timed up and go test and had poorer Oswestry disability index scores compared with the non-spinal sarcopenia subgroup. Regarding patients with degenerative spinal diseases, studies have indicated that the pelvic tilt angle is the sagittal parameter most closely related to skeletal muscle mass [45]. Skeletal muscle mass might be a crucial factor related to the posterior inclination of the pelvis in symptomatic patients with spinal conditions, especially cervical spine disease. Kumagai et al. assessed the association between cervical disc degeneration (CDD) and muscle strength [56]. They observed a significant negative correlation between CDD and trunk strength in both sexes, handgrip strength in men, and leg strength in women. The results revealed that age was the strongest factor independently associated with CDD in both sexes, and the effect of age could be decreased by increased limb and trunk muscle strength.

**Figure 1:**
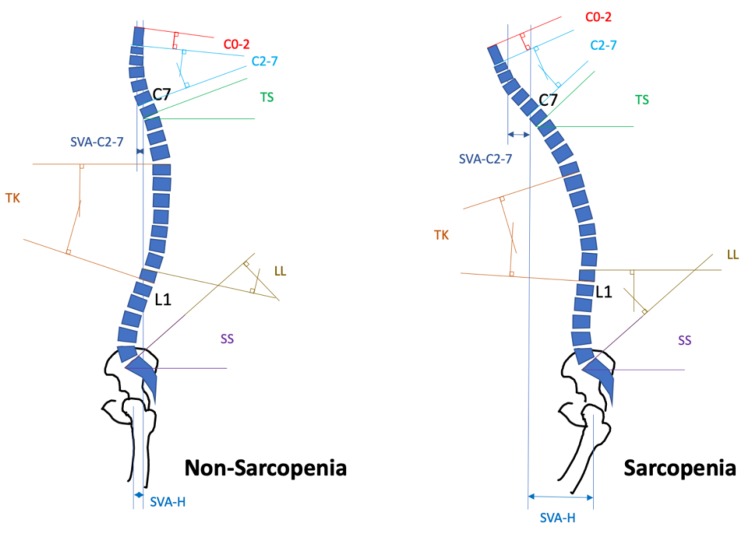
Schematic images comparing the postures of patients with a spinopelvic mismatch in spinal sarcopenia and non-spinal sarcopenia groups. Cervical parameters include C2-7L, cSVA, T1S, and C0-2. Lower extremity parameters include TK, LL, SS, and SVA-Hip. C2–C7L: C2–C7 Cobb angle; cSVA: C2–C7 sagittal vertical axis; T1S: T1-slope; C0-2: C0–C2 angle; TK: thoracic kyphosis; LL: lumbar lordosis; SS: sacral slope’ SVA-H: sagittal vertical axis to the center of hip.

Ignasiak et al. constructed a musculoskeletal multibody model of the thoracolumbar spine to investigate the effects of muscle aging and spinal sarcopenia on muscle recruitment patterns and spinal load [57]. The results demonstrated that the normal aging of muscle had little influence on spinal loads and provoked muscular activities, while severe sarcopenia increased the compression/shear loading of the spine. Furthermore, studies have demonstrated that patients with spinal sarcopenia exhibit insufficient compensation of the thoracic spine, leading to a larger thoracic kyphosis (TK) with sagittal vertical axis (SVA) distance and a smaller cervical lordosis (C2–C7 Cobb angle) and lumbar lordosis (LL) compared with patients without spinal sarcopenia (Figure 1) [58,59]. In fact, SVA-C2-C7/hip/ankle (sagittal vertical axis to the center of C2–C7/hip/ankle) were noted to be larger in patients with spinal sarcopenia, whereas the SVA distance of the knee remained the same to compensate for the spinopelvic alignment. In addition, T1 and sacral slopes were larger in patients with spinal sarcopenia. Based on the aforementioned evidence, it can be inferred that spinal sarcopenia is significantly associated with various spinal disorders including DLS, CDD, and LSCS, and that through measuring the sarcopenia-related spinal alignment, the severity of the spinal sarcopenia can be objectively evaluated.

## 3. Image Detection Methodologies for Spinal Sarcopenia

The muscle quantity of patients with spinal sarcopenia can be measured using several techniques, such as dual-energy X-ray absorptiometry (DXA), bioelectrical impedance analysis (BIA), magnetic resonance imaging (MRI), and computerized tomography (CT) [60]. Tuscle quantity is determined by total body skeletal muscle mass (SMM), appendicular skeletal muscle mass (ASM), or muscle CSA [61]. In addition to routine bone mineral density assessments, studies have revealed that DXA can be used to estimate ASM by using the bone- and fat-free mass of the arms and legs [62,63,64,65]. For muscle mass quantification, the absolute level of SMM or ASM can be adjusted for body size using various approaches, such as height squared (ASM/height^2^), weight (ASM/weight), or body mass index (ASM/BMI) [66]. However, apart from the issue of ionizing radiation from DXA, the measurement of non-fat tissue mass can be performed using DXA based on hydration status and edema [67,68].

**Figure 2:**
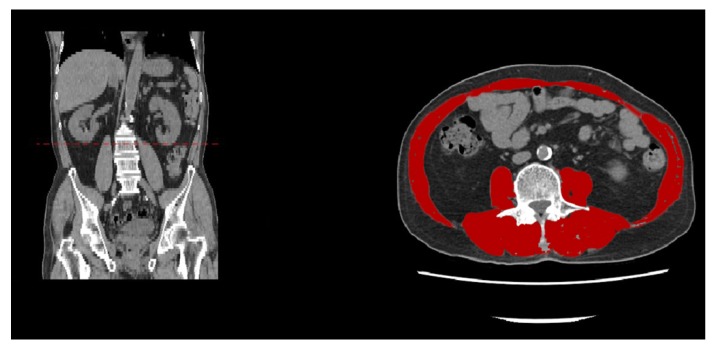
Computed tomography image of the third lumbar vertebral level. The following skeletal muscles are outlined in red: psoas, paraspinal, transverse abdominal, external oblique, internal oblique, and rectus abdominis muscles.

Some researchers recognize MRI and CT to be the most accurate imaging methods for assessing the muscle mass, CSA, muscle quality, and intramuscular adipose tissue [69]. The methods of assessment include third lumbar vertebra imaging (Figure 2) and mid-thigh muscle measurement [70,71]. Even though CT and MRI approaches have not yet met internationally accepted clinical standards, the quantification of total lumbar muscle with CSA has been widely applied. The advantage of the total lumbar muscle CSA-based approach includes the objective representation of different muscles, such as psoas, erector spinae, quadratus lumborum, transverse abdominis, etc. [72]. In 2008, one study demonstrated a high correlation between the SMM assessed by DXA and CSA at the level of the third lumbar vertebra, obtained using CT [73,74]. The study confirmed that L3 is the ideal location for CT-based sarcopenia measurements. Notably, this imaging method has been widely used in patients with catabolic illnesses to detect low muscle mass and predict prognosis [75,76]. Furthermore, when L3 measurement is not available, the alternative levels (in order of preference) are L2, L4, L5, L1, T12, T11, and T10 [77]. The other imaging approach related to sarcopenia is mid-thigh muscle measurement [78]. Mid-thigh imaging can be performed using either MRI or CT. Several researchers have recognized mid-thigh muscle measurement as an excellent predictor of whole-body SMM with a high sensitivity to change [79,80]. Schweitzer et al. revealed that areas at the mid-thigh provided better evidence than lumbar muscle areas for assessments of total skeletal muscle volume [81]. In addition, studies have supported the fact that a reduction in the mid-thigh muscle CSA obtained using a CT scan is better than a low BMI for predicting mortality in patients with chronic obstructive pulmonary disease and their physical performance after hip fractures [82,83]. In addition, Ochi et al. suggested that because of a stronger relationship between the thigh muscle CSA and body weight than body height, the thigh muscle CSA should be corrected using body weight (CSA/weight) as another sarcopenic index [84].

Furthermore, CT measurements of psoas muscle thickness have been used to assess sarcopenia and predict morbidities in certain patients with conditions (e.g., colorectal cancer, ovarian cancer) and those undergoing certain treatments (e.g., colorectal surgery, lung transplantation) [85,86,87]. This measurement is sometimes obtained at the L3 level on the first image in the craniocaudal direction, wherein the two transverse processes are visible [88,89]. Nevertheless, the measurement of the psoas muscle remains controversial. Some researchers have argued that the psoas muscle is not representative of overall sarcopenia because it is a minor muscle [71,72,90]. In 2016, Morrell et al. conducted an observational study to determine whether the measurement of psoas or paraspinal muscles at the same L4–L5 axial slice for abdominal fat measurement could be feasible. The results demonstrated that the psoas muscle provided a better measure of whole-body muscle mass compared to the paraspinal muscle, although it was slightly inferior to mid-thigh measurement [70]. Nevertheless, the application of psoas muscle measurement requires extensive research to evaluate its validity.

Notably, CT and MRI provide details on specific muscles, adipose tissues, and organs which are not provided by DXA or BIA. However, owing to the absence of ionizing radiation and a lower risk of cancer, MRI is considered more advantageous than CT. Meanwhile, due to high equipment costs, the lack of portability, and the requirement for highly-trained personnel to use the equipment, CT and MRI are not used as commonly as DXA and BIA [91]. Hence, future measurement techniques must be cost-effective, standardized, and repeatable by practitioners in various clinical settings and across different patient populations [92,93].

## 4. Impact of Spinal Sarcopenia on Surgical Interventions for Spinal Disorders

The number of lumbar spinal surgeries performed on patients with spinal sarcopenia increases in nations as their populations age. A retrospective observational study analyzed the relationship between spinal alignment and SMM on clinical outcomes after surgery in patients with lumbar spinal stenosis [94]. It suggested that postoperative outcomes were inferior in patients with sarcopenia. Moreover, both preoperative reduced limb muscle mass and posterior pelvic tilt were recognized as predictive factors for postoperative lower back pain. In addition, another comparative study from Japan demonstrated the negative effect of sarcopenia on clinical outcomes after lumbar spinal surgery [95]. Regarding the measurement of sarcopenia, an ambispective study from Canada indicated that sarcopenia measured using normalized total psoas area did not predict acute care complications in elderly patients undergoing simple lumbar spine surgery for degenerative spinal diseases. The modification of the core abdominal musculature because of underlying degenerative spine disease could have precluded the risk stratification based on this measurement [96].

Symptomatic vertebral fractures, which are the most common type of fractures among older adults, are associated with considerably high morbidity, excess mortality, and health and social service expenditure. Retrospective studies have examined the association between vertebral fractures and trunk muscles that are intimately tied to spinal loading and function, and revealed that individuals with vertebral fractures have more fat infiltration in their trunk muscles, low trunk extension strength, and altered muscle activation patterns [97]. Furthermore, in terms of adjacent segment disease (ASD) after lumbar fusion, one study compared the relationship between ASD and lean muscle mass. The result suggested that patients with ASD had a smaller lean muscle mass, a lower ratio of functional CSA to total CSA, and a lower SMI of functional CSA of the paraspinal muscle group on preoperative MRI compared with control patients [98].

In addition to the effect of spinal sarcopenia on lumbar surgery outcomes, several studies have evaluated the effects of spinal sarcopenia on the sagittal alignment of the cervical spine after cervical laminoplasty [99,100]. The results revealed that, compared with patients without sarcopenia, the C2-C7 SVA was greater and the postoperative outcome was worse after cervical laminoplasty in patients with sarcopenia. Therefore, it is imperative to evaluate sarcopenia before cervical laminoplasty, because it may affect both postoperative cervical alignment and postoperative outcomes. In patients with disseminated cancer, age-related muscle loss may worsen because of cachexia and poor nutritional intake [101]. Moreover, in patients with spinal metastasis, the decision to perform decompressive surgery is mainly based on a functional assessment, which might not be an appropriate indicator of life expectancy. Therefore, Gakhar et al. investigated the association between the muscle mass and 1-year survival of patients with metastasis. The results revealed that the mortality rates at 1 year were significantly higher among patients in the lowest quartile of muscle mass than in those in the highest quartile. Hence, an evaluation of lean muscle mass may help surgeons to make appropriate treatment decisions for patients with spinal metastases.

## 5. Conventional Treatment of Spinal Sarcopenia

### 5.1. Nutrition Strategy and Antiinflammatory Medication

**Figure 3:**
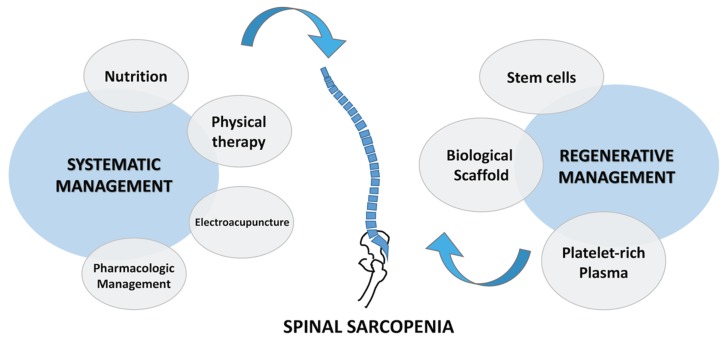
Conventional and regenerative management of spinal sarcopenia. PRP: platelet-rich plasma.

Spinal sarcopenia is treated using a combination of conventional treatment and regenerative management (Figure 3). Conventional management requires a combination of the sufficient consumption of fatty acid and high-quality protein, use of physical exercise, and administration of anti-inflammatory medication. Treatment in the advanced stage involves the prevention of complications of spinal sarcopenia, such as intensified loss of skeletal strength, function, and mass. Therefore, most evidence emphasizes the consumption of essential high-quality dietary items containing antioxidants, proteins, and vitamin D. Furthermore, a study on rehabilitative loading after disuse in an aging mice model suggested that antioxidants such as resveratrol, β-hydroxy-β-methyl butyrate, and green tea catechins improve satellite cell functions [102]. The conservative management of spinal sarcopenia requires a nutritional strategy comprising the sufficient intake of high-quality protein and fatty acids, engagement in physical exercise, and administration of anti-inflammatory medications [91,103]. Therefore, it is better to prevent the progressive loss of SMM, strength, and function before spinal sarcopenia-related complications occur rather than attempt restoration at an older age [104]. Notably, the evidence thus far has highlighted the potential importance of a high-quality diet that ensures the sufficient intake of protein, vitamin D, and antioxidant nutrients [102,105].

### 5.2. Physical Therapy and Resistance Excercise

The development and progression of spinal sarcopenia are complex and multifactorial. Notwithstanding, a growing body of evidence indicates that physical activity can decrease the rate of skeletal muscle loss and impaired function as well as maintain lean body mass [106]. A recent study reported that physical activity decreased lean body mass and increased fat mass but that body weight remained stable. Similarly, earlier studies confirmed changes in body composition and revealed that these changes could not be identified using the body mass ratio [107]. Some longitudinal studies have reported that approximately 70% of people perform moderate to intense physical exercise for approximately 60 to 90 min per week, such as walking, swimming, cycling, running, and playing [108]. Notably, such types of physical activity meet the standard levels of physical activity recommended by the American Heart Association for cardiovascular exercise, but they are not effective in reducing fat mass or maintaining lean mass [109]. Nevertheless, some studies have reported that a higher level of physical activity, which involves resistance exercise, can slow the loss of skeletal muscle oxidative capacity and the onset of spinal sarcopenia [110].

**Figure 4:**
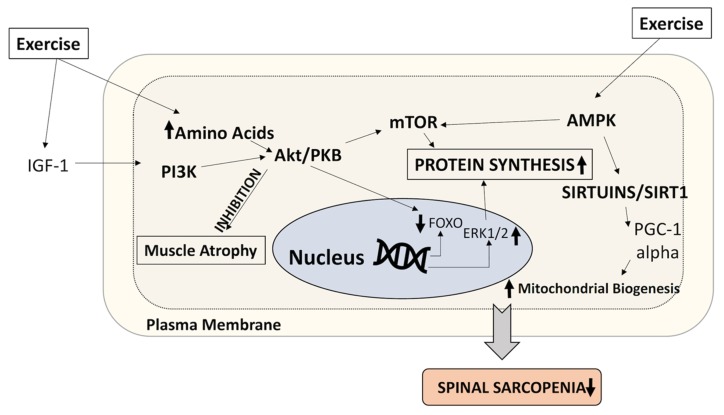
Mechanistic insights into spinal sarcopenia. During aging, the activities of Akt, IGF-1, and AMP-activated protein kinase (AMPK) are reduced. During exercise, increased amino acid levels induce the activity of these components to increase protein synthesis and mitochondrial biogenesis, thereby reducing spinal sarcopenia.

Notably, resistance exercise improves muscle mass, size, and strength in middle-aged and older adults, even those with a history of fractures. Several forms of resistance exercise (e.g., free weights, weight machines, resistance bands, and the use of body weight) are available according to the tolerance and ability of adults, such as their pain endurance [111]. The WHO recommends that individuals aged 65 years or older require muscle strength exercise involving the major muscle groups and that such exercises must be performed at least two days per week [112]. Resistance exercises have proved beneficial to skeletal muscles when they are performed at least twice a week, increased progressively over time, and performed with a high mechanical load, targeting the larger muscle groups of the hip and spine [113]. Resistance exercises can stimulate the muscle protein synthesis involved in muscle contraction. Some studies have demonstrated that resistance exercises cause muscle hypertrophy by promoting an increase in growth factors such as insulin growth factor, which stimulates the PI3K-Akt-mTORC1 signaling pathway to stimulate muscle protein synthesis (Figure 4) [113,114]. However, limited evidence is available regarding the resistance exercise-induced mechanical transduction of muscle protein synthesis in spinal sarcopenia.

### 5.3. Aerobic Training

Aerobic training is beneficial in slowing weight gain and maintaining metabolic health. This decreases inflammation and oxidative stress, increases insulin sensitivity, and improves the blood cholesterol profile [115]. Aerobic exercise produces numerous ATPs in the mitochondria of skeletal muscles and maintains metabolic regulation, aerobic capacity, and cardiovascular function [116]. Moreover, it aids the synthesis of muscle protein through inducing mitochondrial biogenesis, restoring the expression of catabolic genes, and restoring mitochondrial metabolism.

Nonetheless, regarding physical exercise, studies have indicated that although aerobic training plays a crucial role in improving cardiometabolic health, resistance training is probably the optimal approach for delaying spinal sarcopenia [117]. In addition, another study confirmed that exercise intervention proved to be safe and effective for reversing functional and cognitive decline among hospitalized older adults [118]. Concerning spinal sarcopenic patients with diabetes and obesity, studies recommend using the Rapid Geriatric Assessment tool and starting secondary prevention as soon as possible [119,120,121,122]. Overall, exercise training appears to ameliorate mitochondria-associated problems and improve spinal sarcopenia.

### 5.4. Electroacupuncture

Electroacupuncture is a treatment that provides stimulation through acupuncture needles by using a low-frequency microcurrent; it is an alternative technique for managing spinal sarcopenia. According to the Takoaka et al. study in a mouse model, the electroacupuncture treatment suppressed myostatin expression, which resulted in a satellite cell-related proliferative reaction and skeletal muscle repair [123]. The counteraction of diabetes or chronic kidney disease-induced skeletal muscle atrophy by increasing IGF-1 has also been demonstrated in other mouse model studies [124,125]. However, one clinical trial evaluated the effects of acupuncture based on the muscle strength and inflammatory markers of IL-6, IL-10, and TNF-α cytokines in older adults with sarcopenia and observed that the intervention protocol did not result in significant differences in the evaluated population [126].

### 5.5. Pharmacologic Management

In terms of pharmacologic treatment, selective androgen receptor modulators and antimyostatin antibodies are potential stimulators of muscle anabolism [127]. Myostatin inhibition is recognized as a most interesting therpaeutic strategy for spinal sarcopenia compared to resistance training with amino acid supplementation [128]. A proof-of-concept, randomized phase 2 trial revealed that thehumanized monoclonal antibody LY2495655, a myostatin inhibitor, could increase lean muscle mass and could therefore improve functional measures of muscle power [129]. In addition, testosterone, angiotensin-converting enzyme inhibitors, and ghrelin-modulating agents are being investigated as potential agents to treat spinal sarcopenia [130]. Studies have confirmed that testosterone supplementing in large amounts and at low frequency may effectively improve muscle defects during aging [128,131]. Despite being a potential regulator of muscle mass, no positive effect of IGF-I has been demonstrated, which might be attributed to its local resistance [128]. Moreover, a recent study revealed that fermented soybeans may improve glucose tolerance, along with providing a significant reduction in the expression of inflammatory factors related to immune senescence in the skeletal muscles of middle-aged obese rats [132]. Furthermore, an animal trial revealed that urocortin II, a corticotropin-releasing factor 2 receptor (CRF2R)-selective peptide, increased the SMM of mice with normal muscles. Hence, for patients with skeletal muscle wasting diseases, such as age-related muscle loss or spinal sarcopenia, CRF2R-selective agonists may be a treatment option [133]. Nonetheless, such pharmaceutical agents are still under investigation, and more evidence is needed to assess their benefits.

## 6. Regenerative Therapeutic Approaches for Spinal Sarcopenia

### 6.1. Stem Cells

In recent years, several regenerative therapies for spinal sarcopenia have been developed, such as stem- or progenitor-cell-based approaches as well as biologic scaffolds [134]. In terms of stem-cell-based strategies, various animal studies have evaluated different cells for skeletal muscle repair, including satellite cells, muscle-derived stem cells, and embryonic stem cells in mice [135,136,137,138]. Sarcopenia associated with the loss of functional contractile myofiber units has been investigated by using stem cells to repopulate the satellite cells that stimulate muscle protein synthesis or myogenesis [139]. Some preclinical studies have reported promising outcomes from stem cell-based therapies that might not only circumvent economical and technical issues but also regulate obstacles preventing their widespread adoption as a therapy for sarcopenia [140]. Satellite cells are obligated to the myogenic lineage, but other populations of neighboring muscle cells with multilineage potential have been observed to be of help in muscle repair [141]. Muscle-derived stem cells are not terminally obligated to the myogenic lineage and have a myogenic, osteogenic, and chondrogenic differentiation potential. Transplanting muscle-derived stem cells in areas of severe muscle injury resulted in decreased fibrosis and improved muscle repair. Although muscle-derived stem cells have been demonstrated to have benefits in muscular dystrophy models, there is still a lack of evidence for their use in acute muscle injuries [142]. Stem cell therapy is employed for treating sarcopenia either indirectly in signaling pathways or directly in the formation of new myofibrils. Stem cell transplantation improves the muscle contractile function and increases the stem cell niche population in the host muscle. Some studies have provided evidence of stem cell activation and the subsequent repair of injured muscles after acute injuries, but studies using stem cells for transplantation in clinically relevant muscle injuries are lacking [143,144]. Nonetheless, various cells have been identified that do not possess myogenic differentiation potential but can indirectly support regeneration, such as mesenchymal stem cells (MSCs), which have mutidifferentiation potential and can be isolated from sources such as adipose tissue, gingiva, bone marrow, and the umbilical cord [145]. In addition, MSCs can manipulate the function of other cells through endocrine mechanisms or even paracrine signaling [146]. Moreover, they can produce several immune modulators which may exert a beneficial effect on patients with spinal sarcopenia [147]. However, the difficulty of isolating and expanding them in culture as well as poor engraftment efficiency are some inevitable issues that must be solved [134]. In a seminal study, Fry et al. demonstrated that the lifelong reduction in satellite cells did not accelerate sarcopenia or affect the maintenance of muscle size or fiber type composition in aging mice [148]. In general, stem cell therapy was advantageous in regulating muscle synthesis by differentiating into various cell lineages. Based on many studies that have investigated with mechanistic insight muscle repair in mice [135,136,137,138,148], it is recognized to play an important role in treating spinal sarcopenia in the future.

### 6.2. Biologic Scaffolds

**Figure 5:**
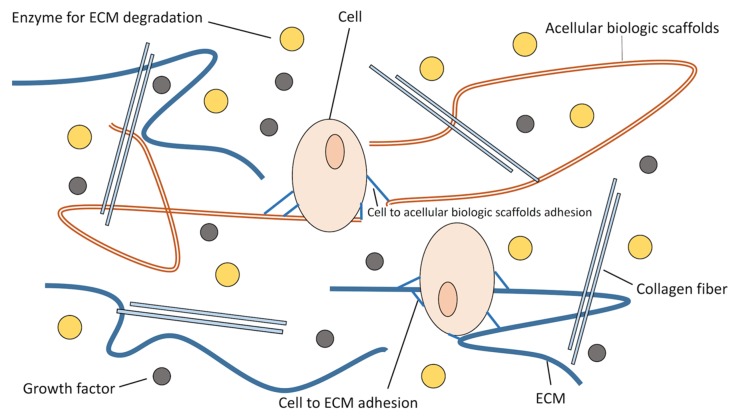
Natural environment of the ECM. The ECM provides nutriment to cells, signaling proteins responsible for intercellular communication, and mechanical support. ECM: extracellular matrix.

During aging or in patients who have experienced traumatic incidents, tumor ablations, or pathological states that lead to the destruction of connective tissue and basement membranes, the complete regenerative program can be affected by fibrotic tissue formation, thereby causing the functional impairment of the skeletal muscle and spinal sarcopenia [149,150]. This impairment is considered to be related to the loss of inherent signals that assist in skeletal muscle regeneration in healthy tissues. For example, regarding nature’s ideal microenvironment, studies have suggested that the implantation of acellular biologic scaffolds, composed of mammalian extracellular matrix (ECM), can improve strength and function as well as promote the site-appropriate remodeling of traumatized skeletal muscle in both humans and rodents [151,152]. ECM is essential to correcting the parallel alignment of myofibers and maintaining mechanical properties for tissue functionality (Figure 5) [153]. Overall, biologic scaffolds may represent a possible therapeutic option for optimizing the muscular microenvironment in the acute muscle injury of high risk patients to maintain muscle function and prevent spinal sarcopenia.

### 6.3. Platelet-Rich Plasma

Platelet-rich plasma (PRP) has repair and regeneration potential in muscle injuries [154]. Several trials have demonstrated the faster healing, less swelling, and decreased time of return to play in patients with muscle strains [155,156,157]. In an experimental rat model, the mRNA level of proinflammatory cytokines IL-1β and TGF-β1 was increased after PRP treatment [158]. Moreover, this phenomenon modulates the expression of several myogenic regulatory factors, such as MyoD1, Myf5, and Pax7, as well as the muscular isoform of insulin-like growth factor 1 (IGF-1Eb). The modulation of these molecular mediators contributes to effective muscle regeneration [158]. Nonetheless, more human trials are required to clarify the relationship between PRP treatment and spinal sarcopenia.

The aforementioned studies have demonstrated the effect of spinal sarcopenia on different spinal surgeries and the management of spinal sarcopenia. Hence, it is imperative to control spinal sarcopenia well both preoperatively and postoperatively. However, more studies are required in the future that focus on how to manage spinal surgery-related adverse events, such as instrumentation failures and positioning-related complications, to offer clarification on several aspects related to spinal sarcopenia.

## 7. Conclusions

Spinal alignment may be influenced by an imbalance between the extensor and flexor back muscles and provides an objective measurement to evaluate the severity of spinal sarcopenia. Image detection methodologies have evidenced that L3 is recognized as the most ideal site for CT-based sarcopenia measurements. Spinal sarcopenia patients usually have inferior outcomes after different surgeries. Although nutrition supplementing, physical therapy, and medications have been proven to be effective, regenerative therapeutic options seem to be promising owing to their repair and regeneration potentials. Therefore, future research should focus on providing a clear understanding of the pathogenic mechanism of spinal sarcopenia and its relationship with other spinal disorders to develop therapeutic modalities in conjunction with surgical and regenerative approaches.
